# Potential regenerative rehabilitation technology: implications of mechanical stimuli to tissue health

**DOI:** 10.1186/1756-0500-7-334

**Published:** 2014-06-03

**Authors:** Colleen L McHenry, Jason Wu, Richard K Shields

**Affiliations:** 1 Department of Physical Therapy & Rehabilitation Science, Carver College of Medicine, University of Iowa, 1-252 Medical Education Building, Iowa City, IA 52242-1190, USA

**Keywords:** Vibration, Mechanical oscillation, Compression, Mechanical load, Spinal cord injury

## Abstract

**Background:**

Mechanical loads induced through muscle contraction, vibration, or compressive forces are thought to modulate tissue plasticity. With the emergence of regenerative medicine, there is a need to understand the optimal mechanical environment (vibration, load, or muscle force) that promotes cellular health. To our knowledge no mechanical system has been proposed to deliver these isolated mechanical stimuli in human tissue. We present the design, performance, and utilization of a new technology that may be used to study localized mechanical stimuli on human tissues. A servo-controlled vibration and limb loading system were developed and integrated into a single instrument to deliver vibration, compression, or muscle contractile loads to a single limb (tibia) in humans. The accuracy, repeatability, transmissibility, and safety of the mechanical delivery system were evaluated on eight individuals with spinal cord injury (SCI).

**Findings:**

The limb loading system was linear, repeatable, and accurate to less than 5, 1, and 1 percent of full scale, respectively, and transmissibility was excellent. The between session tests on individuals with spinal cord injury (SCI) showed high intra-class correlations (>0.9).

**Conclusions:**

All tests supported that therapeutic loads can be delivered to a lower limb (tibia) in a safe, accurate, and measureable manner. Future collaborations between engineers and cellular physiologists will be important as research programs strive to determine the optimal mechanical environment for developing cells and tissues in humans.

## Background

Vibration and compressive loads are mechanical stimuli that have a powerful influence on biological tissues. Recent studies in animal models demonstrate that certain types of mechanical load regulates bone [1], fat [2,3], skeletal muscle [4,5], and nerve tissues [6]. In addition, it is also well known that “over exposure” to mechanical stimuli is damaging to tissues [7-9]. With the emergence of regenerative medicine in tissue repair, rehabilitation specialists must understand the correct type and dose of mechanical stress that promotes cell survival and cell proliferation in bone, cartilage, ligaments, and muscle. However, to our knowledge, there is no technology that directs specific types of mechanical stimuli to limbs of humans. A method to study mechanical stimuli in humans is necessary to guide future research to determine optimal rehabilitation prescriptions. The importance is underscored as multi-potent adult stem cells are harvested and implanted after surgery, injury, disease, and paralysis as regenerative medicine advances. Our long term goal is to establish the extent to which various types of mechanical stimuli optimally influence the regenerative capacity of cells in humans. In this technological report, we present an innovative technology that may assist in determining the impact of mechanical stimuli of human tissues and discuss the importance of a partnership between engineers, bioscience researchers, and rehabilitation specialists.

The underlying need to study the value of therapeutic stress in humans is well grounded in the literature. For example, Wolff’s law supports that bone tissue (osteocytes) exposed to high loads triggers osteogenesis [10]. Subsequent studies verified that exerting high strain in a dynamic fashion to bone tissue was more effective than delivering a sustained strain [11,12]. For many years, the dynamic delivery of high stress to bone was considered the primary mechanical method to up-regulate osteogenesis [13-15]. However, more recently, low amplitude vibration stimuli, in the absence of high mechanical loads, were equally effective at up-regulating bone development in mice [5,16-19]. Indeed, regular mechanical stress promotes a healthy environment for bone [1], fat [2,3], skeletal muscle [4,5], nerve tissue [6], and cartilage (articular, menisci) [20,21] in animal or reduced preparations in the laboratory. Translating these findings into human studies has been hampered by the lack of a capacity to dynamically deliver high passive loads and/or low vibration either independently or in various combinations with or without muscle activation (electrically or volitionally).

The dose of various mechanical loads has not been carefully examined. For example, most studies evaluating vibration deliver the load to the entire animal [5,16,19,22-24] or human [25-30] which limits the ability to understand adaptive effects of localized vibration directly on tissues (muscle and bone). This point was emphasized when whole body vibration of mice had a systemic increase in bone density [5,18,19] and decrease in whole body adipogenesis [3]. The direct effect of the vibration stimuli on bone tissue was confounded by vestibular [31] and/or endocrine system [32] mechanical activation.

The purpose of this technological report is to present a novel method to introduce localized compressive loads and/or vibration into the limbs of humans. The accuracy, repeatability, transmissibility and safety of the instrument will be presented in this report. Future studies are recommended using technology that will assist in better understanding the impact of mechanical stimuli on tissue health. The need for collaborative and inter-disciplinary teams of engineers and cellular physiologists will be emphasized.

## Methods

### Technology development and testing study subjects

Eight individuals with complete paralysis were tested on two occasions to determine the ability to reliably and accurately deliver the mechanical oscillations and loads to the limb of people with spinal cord injury (SCI). A power analysis revealed that 8 participants were required to have power to assess the reproducibility of the system (>80%). Informed written consent was obtained from all subjects prior to participation. All experimental protocols were approved by the University of Iowa Institutional Review Board.

### General description of instrumentation

**Figure 1:**
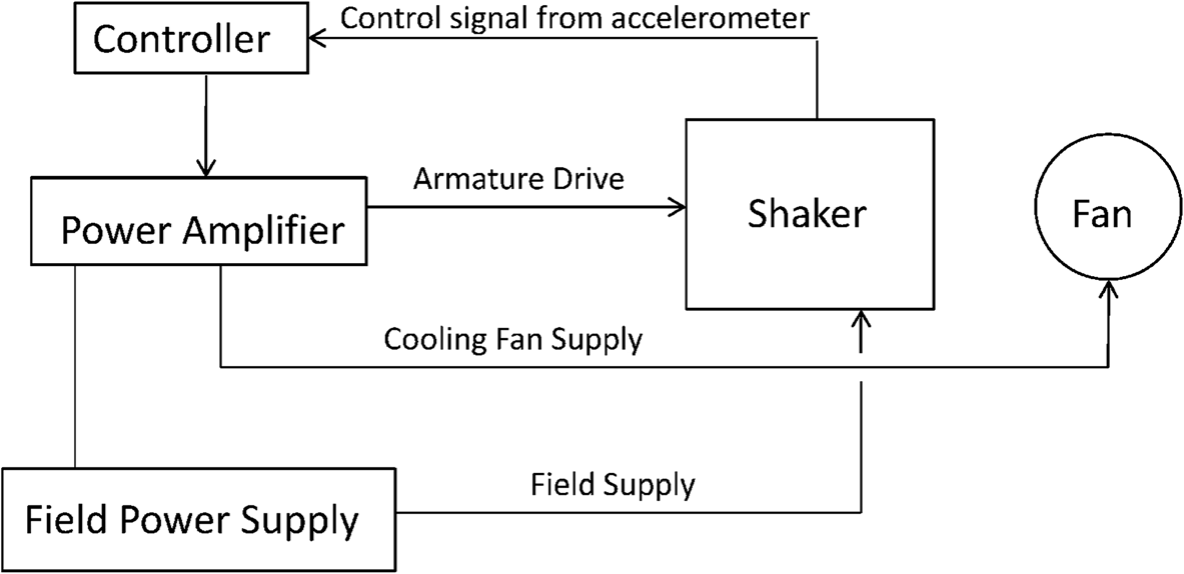
**Schematic of the vibration system.** The power amplifier and field power supply generate power for the system and supply the shaker and the cooling fan. An accelerometer is attached to the shaker and controller creating a feedback loop to control the frequency and magnitude of vibration.

**Figure 2:**
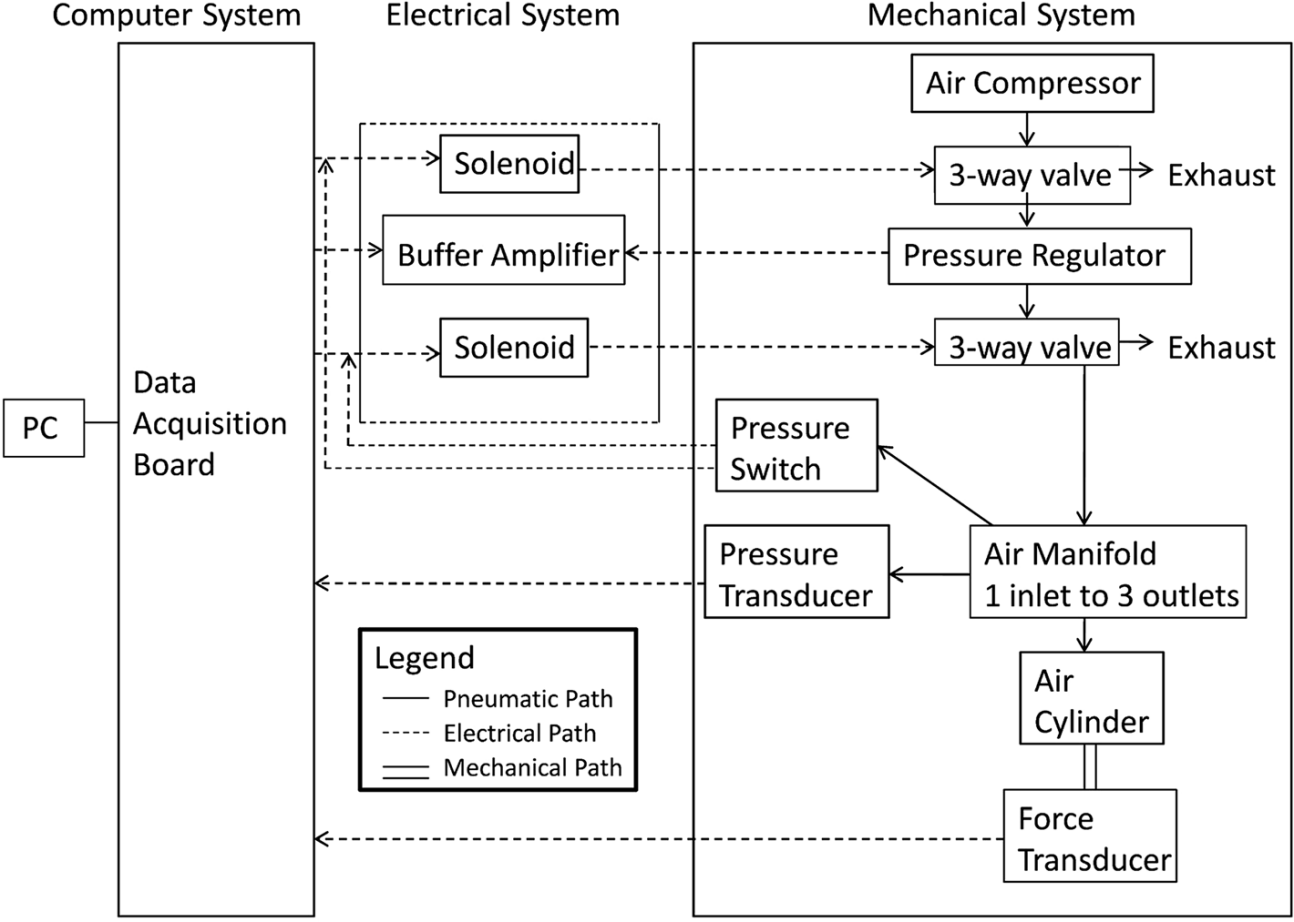
**Schematic of the compression system.** The mechanical portion consists of a series of hardware components which regulate the amount of air pressure delivered to the air cylinder and subsequently the load applied to the human tibia. The electrical system provides power to many of the mechanical components and links them to the data acquisition (DAQ) board. The personal computer (PC) and the DAQ board control the compression system and allow the user to program the compressive system.

A servo-controlled vibration system (Figure 1) consists of five primary components from the Ling Dynamic Systems (Royston, England): PA1000L power amplifier, FPS10L field power supply, V722 shaker, cooling fan, and Laser USB 6.30 controller. The power amplifier and field power supply are connected in cascade and generate the required power for the vibration system. A magnetic field within the shaker is generated from the field power supply while the power amplifier drives the shaker and supplies power to the cooling fan. The cooling fan dissipates the heat generated. An accelerometer is attached to the shaker and connected to the controller, which is directed to the amplifier creating a feedback loop. The vibration frequency in Hertz (Hz) and acceleration in gravitational force of earth (g = 9.81 m/s^2^), respectively, are controlled. The software also allows the user to program multiple loops thereby creating a series of on and off cycles of vibration. The controller is also equipped with an abort button designed to stop the vibration quickly. When providing a mechanical intervention to humans it is important to have built in safety mechanisms in the event of an emergency.We interfaced a custom designed, pneumatically controlled piston that can safely deliver compressive loads to a limb segment either with our without the vibration (Figure 2). The mechanical loading system is driven by a pneumatic compression pump that is controlled by a custom circuit board communicating via the computer interface board. Custom software allows for parameter specification, feedback control, and safety shut down when unwanted loads are inadvertently applied.

The air flow to the limb loading piston begins at the air compressor, a Super Silent DR 500 Air Compressor (Silentaire Technology, Houston, TX). It regulates the air pressure entering the regulator to approximately 552 kPa. The air then passes through a Humphrey 3-way solenoid valve (Skarda Equipment Company, Inc., Omaha, NE). When the solenoid receives 12 Volts from the electrical portion of the system the valve closes and the compressed air remains in the pneumatic system. However, in the absence of power the valve remains open and the air vents to the atmosphere. If the valve is closed then the air continues to the next component, an electrical pressure regulator, T500X Miniature E/P Transducer (Control Air Inc., Amherst, NH). The pressure regulator converts a voltage from a buffer amplifier to a corresponding pneumatic output. The air then moves through a second 3-way solenoid valve and continues to an air manifold. The air manifold divides the air between a pressure switch, 2PSW2SYT5 Pressure Switch (Solon Manufacturing Co., Chardon, OH), a pressure transducer, PT100R13LU2H1131 Pressure Transducer (Turck Inc., Minneapolis, MN) , and an air cylinder, USR-32-1 Pneumatic Cylinder (Clippard Instrument Laboratory, Inc., Cincinnati, OH). The pressure switch is composed of two electrical switches and a diaphragm sensing element. If the pressure is greater than 414 kPa then the circuit is tripped and the loading system shuts down. The pressure switch is one of the safety mechanisms built into the system. The pressure transducer converts the pneumatic input to a voltage that is sent to the electrical portion of the system. The desired air pressure continues into the chamber of the air cylinder causing the piston to move downward. A force transducer, 1210ACK-300 lb Load Cell (Interface, Scottsdale, AZ), and pad are attached in series to the piston and allows pressure and force measurements simultaneously.

**Figure 3:**
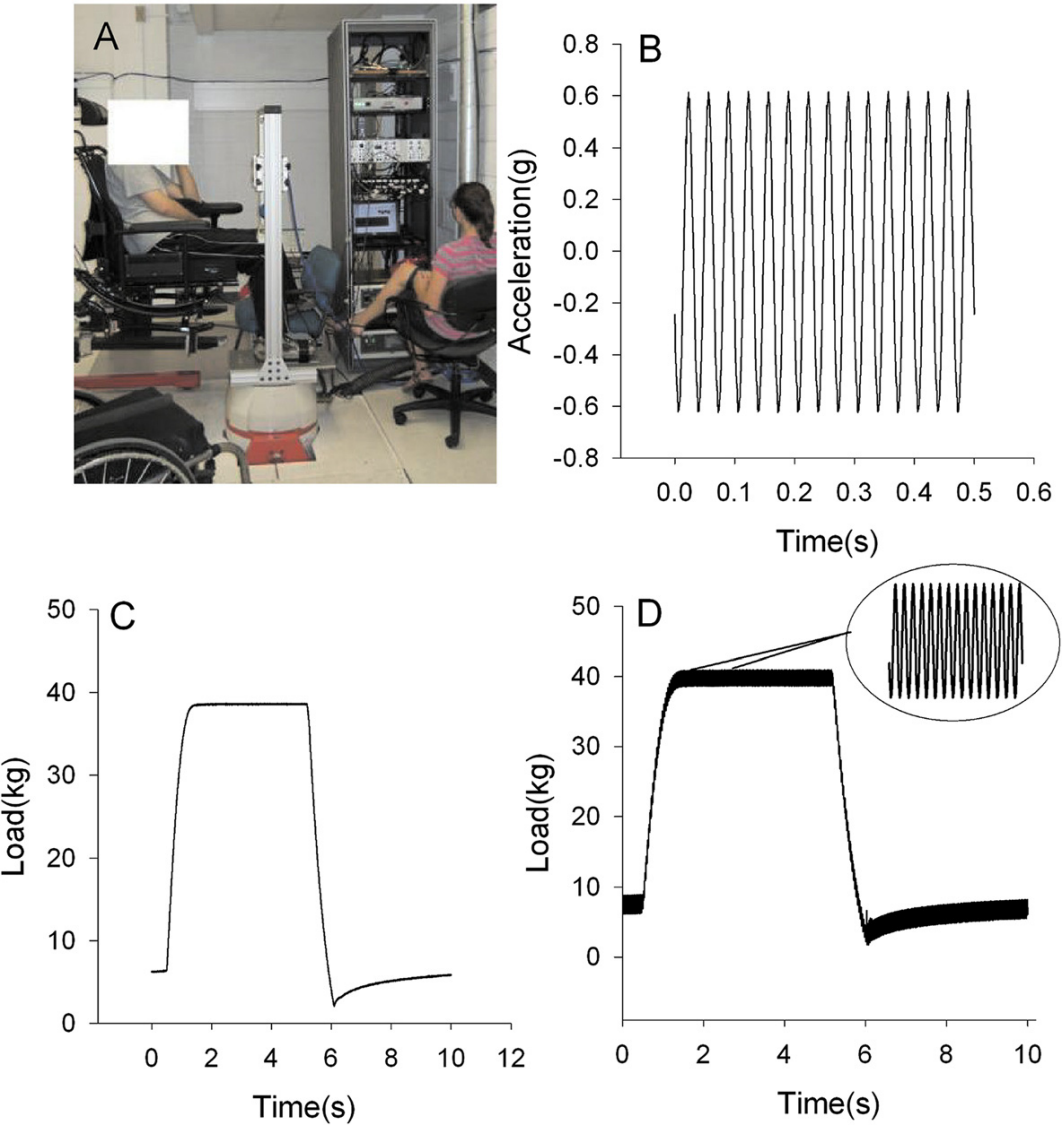
**Vibration and compression system. A)** A participant seated in the adjustable wheelchair with the lower limb secured to custom designed compression frame which is fixed to the vibration shaker. The cabinet rack houses the compression hardware, DAQ board, computer, vibration controller, field power supply, and power amplifier. **B**-**D)** The output of the **B)** vibration, **C)** compression, and **D)** the two systems together was measured for 10 seconds or 1 cycle.

The limb loading system was designed to introduce a vertical compression load to the tibia via a load applied over the top of the femoral condyles (knee) as a percentage of body weight (%BW). A feedback loop was incorporated into the software design written in LabVIEW 8.6 (National Instruments, Austin, TX) to continuously monitor the force and pressure through the transducers and adjust accordingly. The user can define the time that the load is on and off in seconds, the number of cycles, and the magnitude of the force. In addition, data is collected with real-time display of force, pressure, electromyography (EMG), vibration, and other mechanical factors.The apparatus that serves to hold the human limb consists of a custom designed frame that was fabricated and attached to the shaker so that vibration and load can be delivered concurrently (Figure 3). The novelty of this system is that it enables the load to be applied while the entire limb segment receives vibratory stimuli. Thus, during vibration, a force-time impulse may be delivered to the extremity. The frame is made of an aluminum base plate and foot plate connected with T-slot frames. The uprights and cross bar are also made with aluminum struts. Aluminum housing contains the air cylinder and slides within the frame uprights allowing full adjustability for limb length. In addition, a tilt in space chair was welded to a lift that allows any subject, including individuals with paralysis to be positioned correctly into the device.

### Vibration verification and transmissibility testing

We applied an independent external accelerometer, Model 3233A High-Sensitivity triaxial accelerometer (Dytran Instruments, Inc., Chatsworth, CA), to the vibration platform. The Laser vibration software is capable of various vibration parameters. We included settings aligned with those found to be effective in previous studies (0.1 g-10 g at 20–90 Hz) [25-30]. In 2009, Totosy de Zepetnek presented a review of whole body vibration which concluded the optimal vibration parameters for humans have yet to be determined [33]. To test the transmissibility of the vibration signal, the software was programmed for 0.6 g at 30 Hz for 1 minute. The work of Garman and Ozcivici demonstrated the vibration of 0.6 g enhanced the bone of the vibrated limb compared to the contra lateral limb [17,18]. During the vibration, acceleration was collected in all the cardinal directions. The x and y axes were parallel to the platform and the z axis was a perpendicular measure of the acceleration in the vertical direction. A custom MATLAB program (MathWorks, Natick, MA) was written to determine the peak of the acceleration and its frequency content. The peak was defined as the maximum value of the acceleration signal. To determine the frequency of the signal a fast Fourier transform (FFT) was performed. Based on the sampling frequency of 4,000, 32.7680 seconds or 2^17 data points of acceleration data were used for the FFT. This window of data was chosen so the length of data was a power of 2, the recommended length for a FFT.

An accelerometer was attached to the leg, thigh, and head during the vibration protocol of both the vibrated and contra lateral limbs in a single subject. Anatomical locations were defined as tibia tuberosity, distal thigh, and forehead. We defined transmissibility as the ratio of the root mean square (RMS) of acceleration of the anatomical site to the RMS of the acceleration at the mechanical apparatus, consistent with Rubin et al. [34].

$$\mathrm{Transmissibility}=\frac{\mathrm{RM}S_{\mathrm{acceleration}\_\mathrm{body}}}{\mathrm{RM}S_{\mathrm{acceleration}\_\mathrm{platform}}}$$

### Repeatability, linearity, and accuracy testing of limb load

The custom software controls the instrumentation to deliver air pressure to the desired load to the lower leg. The calibration between the air pressure and the delivered force to the limb was determined using five known input pressures (138, 207, 276, 345, 414 kPa). The five input pressures were chosen to calibrate the system. We targeted loads that were able to secure the limb to the device and loads that we previously published to modulate spinal cord activity [35,36]. Ten cycles were collected at each pressure. The accuracy of the limb loading system was measured by determining the linearity, repeatability, and percent error. Linearity was defined as the maximum deviation of the mean difference between the predicted response and the measured load. Repeatability was the maximum difference between measures under the same testing conditions, while percent error was calculated using the following equation, ((measured value-predicted value)/predicted value)*100. Repeatability and percent error were normalized and expressed at percentage of full scale (%FS). Prior to this air pressure validation, the load cell was calibrated. The maximum acceptable error for these three measurement was less than 5%FS.

We delivered limb loads to individuals with complete paralysis to test the reproducibility of the apparatus. Eight individuals underwent two sessions on different days to determine the between day reliability of load delivery to human limbs. Ninety compressive load cycles of 50% of body weight were delivered to one leg of the individuals with paralysis. These five second loading cycles were separated by five second rest periods so ninety cycles took 15 minutes to complete. The peak load was measured after cycles 1, 30, 60, and 90 for each session. The percent difference between days and the intra-class correlation coefficients (ICC) at each time point were calculated (IBM SPSS Statistics Version 19). An ICC > 0.8 indicates that the system has high reproducibility in delivering mechanical load on a day to day basis [37]. Included in this error assessment is the ability to connect the human subject to the mechanical interface system. Any error less than 10% was considered low for the between day reliability assessment.

### Load safety assessment

Since this device is designed to interface with a human tibia, safety is of utmost importance. Although, the vibration parameters (0.6 g, 30 Hz) for this intervention are safe for humans, the system is capable of generating much larger vibration signals (66.3 g, 400 Hz) The shaker parameters were altered so that the maximum acceleration is 6 g and the shaker itself has an over travel protection that limits the peak-to-peak excursion to 11 mm. Finally, the vibration controller is equipped with an abort button that will immediately shut down the system.

The compressive system also has several safety features including an emergency stop switch that removes the load by venting the air to the environment. In addition to a mechanical stop, before starting the compression system, the user must input the cycle time, air pressure, and maximum load. The maximum load is the safety parameter which can be set to ensure that an excessive load for human tibia cannot be inadvertently applied.

To assess the safety of the compression an air pressure of 263 kPa or 445 N was programmed into the system while varying the maximum load. Seven maximum load settings, 423 N, 437 N, 441 N, 445 N, 449 N, 454 N, and 467 N were tested. The force was recorded using custom LabVIEW software written to control the compression system. The effectiveness of the maximum load safety setting was determined by examining the force signal and measuring the peak force delivered.

## Findings

### Transmissibility and quality of vibration signal

**Figure 4:**
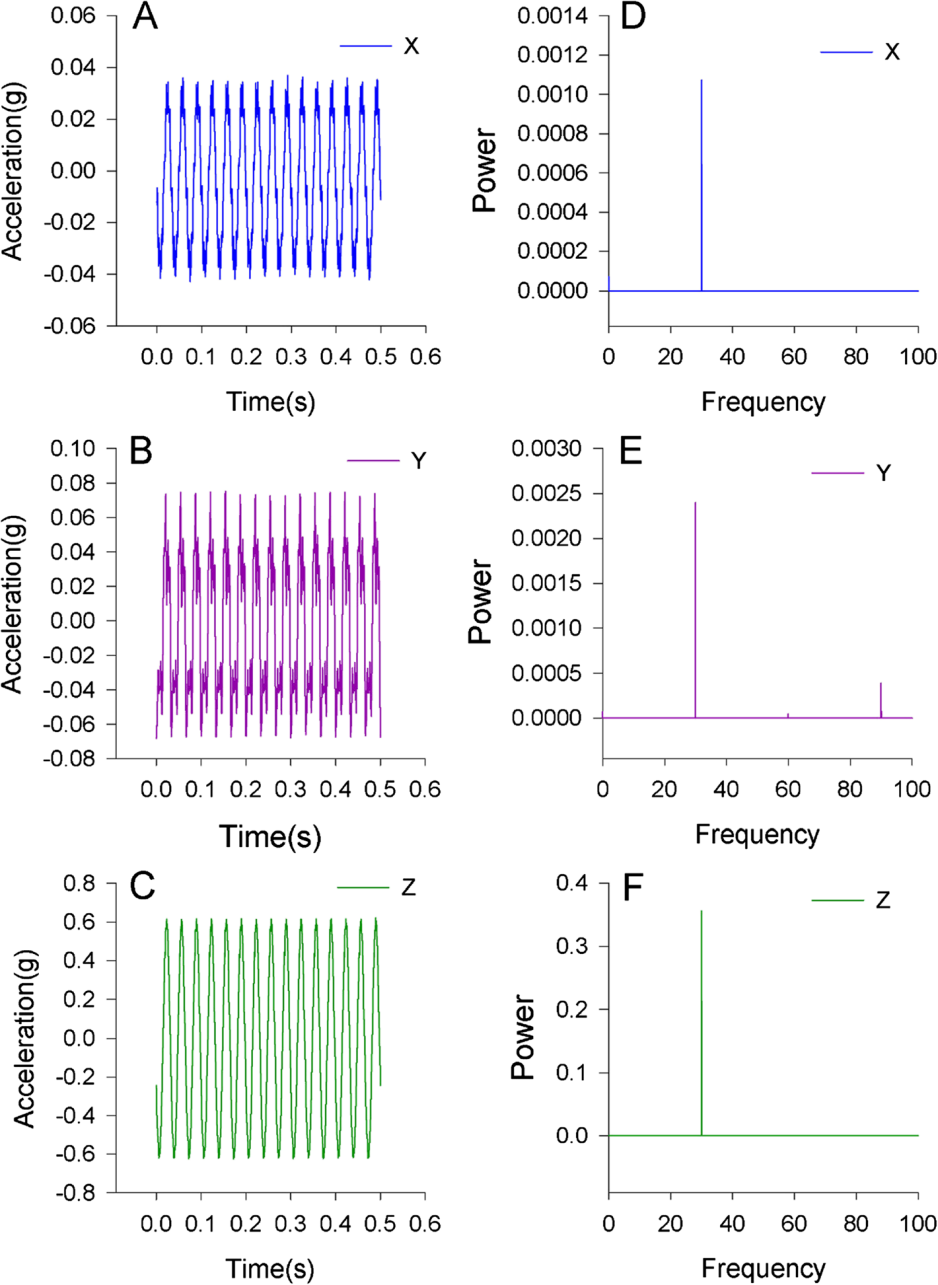
**Acceleration of the vibration platform. A**-**C)** Magnitude of acceleration in the x, y, and z directions are shown. As designed, virtually all of the vibration occurs in the vertical or z direction with minimal acceleration in the axes parallel to the platform. **D**-**F)** Fast Fourier transform of the vibration signal confirms that the frequency content of the vibration is desired frequency of 30 Hz. It also demonstrated that the z-direction contained most of the frequency content.

**Figure 5:**
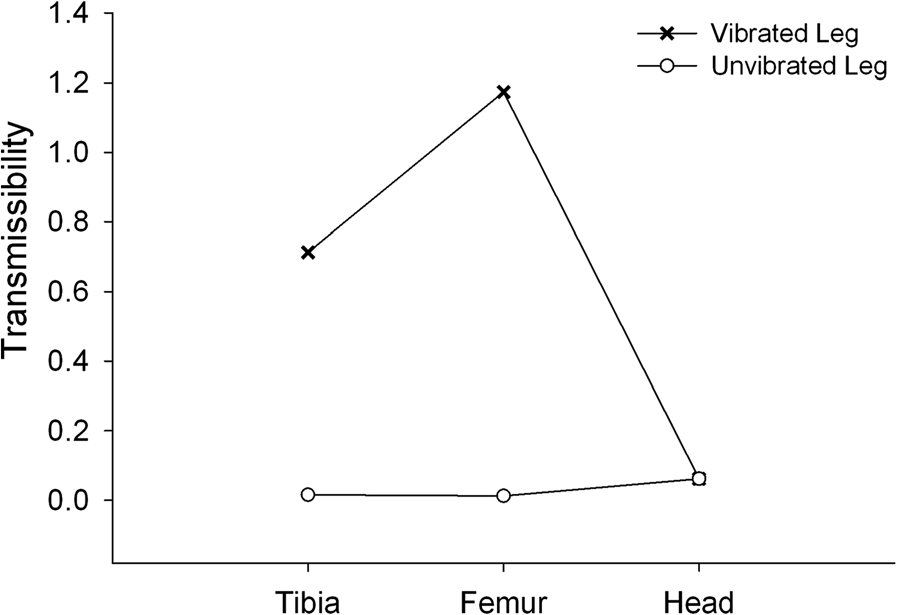
**Transmissibility of the vibration.** The transmissibility of the vibration signal was calculated as a ratio of the anatomical landmark RMS to the RMS of the platform. An accelerometer was place on the tibia and femur of the vibrated and unvibrated leg as well as the head. A transmissibility of 1.0 indicates that the acceleration of the anatomical site is equal to the vibration platform.

At a setting for a 0.6 vertical (z) acceleration and 30 Hz frequency the actual peaks were 0.0406 g, 0.0732 g, and 0.6289 g, for the x, y, and z directions, respectively. There was minimal acceleration in the planes parallel to the vertical platform direction. Through a Fast Fourier Transform we verified that over 98% of the signal power was in the intended 30 Hz frequency domain (Figure 4). Transmissibility, defined as the ratio of vibration amplitude at the anatomical site to the vibration amplitude measured at the shaker, should be equal to 1.0 if there is perfect transmissibility of the vibration to the limb segment. The transmissibility at the tibia and femur were 0.71 and 1.17, respectively. The transmissibility of vibration at the human head and the contra lateral tibia and femur was less than 0.02 (Figure 5). Therefore, the entire system directs the most of the mechanical events specifically to the targeted limb segment.

### Limb load testing results

The linearity, repeatability, and error were calculated at each air pressure was 4%, 1%, and 1%, respectively (Table 1). The between session reproducibility assessment using human subjects was excellent with an intra-class correlation of 0.90 (Table 2). The percent change in limb load never exceeded 7% during between day tests. These data support that total error associated with “setting up” a human subject was low.

**Table 1:** **The compression system performed with a high level of accuracy which is indicated by the linearity**, **repeatability**, **and percent error**

**Pressure (kPa)**	**Linearity (%)**	**Repeatability (%FS)**	**Error (%FS)**
138	3.79	0.54	0.51
207	1.75	0.57	0.42
276	1.26	0.61	0.44
345	0.83	0.69	0.38
414	0.58	0.54	0.32

These metrics were calculated using the 10 cycles at each air pressure (%FS = percent full scale).

**Table 2:** **The data of eight spinal cord injury subjects were used to determine the inter**-**session reliability of the compression system**

**Cycle**	**Change (%) ± SD**	**ICC**
	5.07 ± 2.74	0.917
30	3.43 ± 1.43	0.965
60	6.53 ± 3.98	0.899
90	3.06 ± 2.75	0.965

The difference in force between session at the same time points (after cycles 1, 30, 60 and 90) showed minimal changes and a high intra-class correlation.

### Vibration and limb load safety results

The vibration system consistently shutdown when the acceleration exceeded a 6.1 g, if the platform exceeded 11 mm of displacement, or the user manually pushed the shutdown switch built into the controller. In addition, activating the emergency stop switch consistently aborted the limb loading system by exhausting the compressed air into the environment. To formally test the safety mechanisms under software control, we input a load of 445 N (263 kPa) to the simulated extremity. We then intentionally exceeded the maximum load by programming in loads in excess of the 445 N threshold. The system consistently exhausted the air by the 3-way valve when the 445 N threshold was exceeded. We next set the threshold at 423 N, 437 N, and 441 N and delivered a load of 445 N. Because of a one second delay in the release of pressure, the limb segment received 435 N, 445 N, and 448 N rather than the 423 N, 437 N, and 441 N that were intended. Thus, the safety shut off was effective to within 3% of the intended load.

We had no subjective complaints from any subjects during this testing. There were no tissue areas of reddening or indentations that support that mechanical load of vibration and compression can be delivered concurrently to human tissue.

## Discussion

Currently, there are no existing devices that can provide isolated mechanical loading to a human limb by delivering controlled vibration and/or compression. There are devices which can vibrate [18] or compress [38] the limb of a rodent but neither can deliver vibration and compression simultaneously. Many of the existing devices for humans are commercial vibration platforms that are inherently noisy [39] and typically used for whole body vibration and not localized vibration. The vibration system presented in this paper is servo-controlled and therefore provides a constant vibration using the feedback from the accelerometer to modulate the vibration. Vibration platforms have been widely used in human research; however, prior to the mechanical system presented in this article, there was not a device capable of delivering limb vibration with or without limb compression to an isolated segment.

### Bridging the gap: engineering and bioscience

The primary purpose of this technological report was to present the development of an accurate, controlled, repeatable, and safe mechanical system that would be able to induce localized biological stress to tissues within a limb of humans. Based on our presentation of the findings, we are confident that this system can reliably deliver the stresses within the loads tested based on animal studies and preliminary human reports. Our secondary purpose was to use this report to appeal to the scientific community about the importance of inter-disciplinary teams partnering as cellular therapies are developed in the bioscience laboratories. Our ability to test and learn about the optimal methods to stress tissues is paramount for many new cellular therapies developing today. A brief review of the impact of mechanical stimuli on various tissues will be presented in the subsequent sections.

### Mechanical stimuli and bone tissue adaptation

The relationship between mechanical loads and tissue adaptation is long standing. Wolff [10] and Frost [40,41] demonstrated many years ago that bone tissue is highly mutable and adapts to mechanical stress. In recent years it is well documented that the musculoskeletal system deteriorates in people with SCI [42-46], people on bed rest [47], or people exposed to spaceflight [48,49]. In just two years after paralysis, people with spinal cord injury have 23%, 25%, and 19% less articular cartilage in the patella, medial tibia, and lateral tibia, respectively [50]. Timely mechanical stress reduced the loss of bone by 32% in people with SCI [40,51], which may ultimately be lifesaving [52]. Even secondary systemic complications like renal failure and metabolic syndrome are linked to deteriorating skeletal muscle and bone tissues [53-60].

Low-magnitude whole body mechanical oscillation (0.2-0.3 g, 30 Hz), which would be well tolerated in people who already have osteoporosis, has been shown to attenuate bone loss in women with low bone mineral density [26,61]. Whole body vibration (0.3 g, 45 Hz), at doses similar to that tested in this study, led to 75% increase in trabeculae of the proximal metaphyses of rats [5,19]. Vibration (0.3 g, 30 Hz) of the sheep hind limb showed 34.2% increase in femur bone density [1]. However, only one animal study delivered direct limb segment vibration in-vivo, but showed the tibia had a 88% higher rate of bone formation using the same 0.6 g force demonstrated in the technology presented in this report [62]. Because the vestibular system was likely activated during the weight bearing studies, it is possible that reflexes caused muscle activations that contributed to the tissue changes observed. These studies suggest that understanding the effects of mechanical stress on tissue is complicated and the field may benefit from technologies that isolate these mechanical stresses.

### Mechanical stimuli and cartilage adaptations

Mechanical loading can alter articular cartilage, intervertebral discs, and menisci [63-67]. Knee menisci are particularly susceptible to injury [68] and are often resistant to healing [69]. Cyclic loading and intermittent tensile strain up-regulates VEGF (vascular endothelial growth factor), a gene directly involved with blood vessel formation [70]. Importantly, regular mechanical load reduces inflammation initiated by interleukin-1 following menisci injury [21,71]. A torn porcine meniscus exposed to various mechanical compressive loading conditions (1, 10, or 26% strain, and 4 h/day for 14 days) showed a reduced inflammatory response and repaired mechanical tissue shear strength [21]. The value of cyclic repetitive loads on meniscus health is well documented [72,73]. Importantly, in the absence of natural mechanisms of meniscus tissue repair, regenerative rehabilitation engineers have developed a new scaffold consisting of viable undifferentiated cells that require a healthy environment (optimal stress) to proliferate and differentiate cells [74,75]. Injured meniscus cartilage was merely removed from the knee as little as 25 years ago. Today, the emphasis is in preserving and healing the tissue; however, the effect of controlled dynamic loads with vibration has never been explored in humans with menisci injury or repair. Hence there is a need for technologies to better study the interface between mechanical stimuli and tissue repair in humans.

### Mechanical stimuli and muscle/CNS adaptations

Localized limb vibration modulates several central nervous system and muscle signaling pathways in people with and without spinal cord injury [35,36,76,77]. During single limb segment vibration, the activity of the soleus muscle was suppressed [76]. Vibration caused an 83% reduction in the Hoffmann reflex (H-reflex), but limb load facilitated segmental excitability (decreased H-reflex post activation depression) [35,77]. Likewise, direct vibration over a muscle tendon increased pre-synaptic inhibition of the H-reflex [78-82] and loading (standing) reduces H-reflex post activation depression [83-85]. Recent research has shown that deficiencies in postural control were associated with brain activity during localized vibration of the foot [86].

Vibration platforms for balance control have been reported to cause increased skeletal muscle activity, strength, and power [28,87-89]. These whole body vibration protocols used 2.3 g-30 g at 15–50 Hz, parameters within the range tested in the technology reported in this paper. However, these findings are not supported with direct tendon or muscle vibration; subsequent studies with tendon vibration support a decrease in quadriceps muscle activity and force [90,91]. Some intriguing findings suggest that localized vibration mitigates muscle atrophy during reduced activity [92,93] and regulates certain genes associated with atrophy and synaptic plasticity [4,77]. It may be that the same dose that is optimal for bone is also optimal for cartilage, muscle, and nervous system tissue.

### Mechanical stimuli and stem cell stimulation

An in depth coverage of stem cells is beyond the scope of this technical report. However, a brief summary is warranted. We now know that stem cells require an environment with appropriate stresses to foster survival, proliferation, and ultimately specialization. We also know that vibration input at a 5 g force and 30 Hz frequency caused adult stem cells to differentiate into bone cells [94], and cartilage precursor cells differentiated into cartilage after cyclic mechanical loading (1 Hz, 10% strain rate) [95], similar to the stimuli that we tested in this technical report. Furthermore, recently, chondrocytes were shown to survive longer if they had been exposed to vibratory input and intermittent compressive loading [63,96]. Quiescent satellite cells in skeletal muscle showed enhanced gene regulation for protein synthesis following vibratory input at 30 Hz frequency [4]. Clearly, the degree to which a satellite cell will evolve from the undifferentiated state to the specialized state is under the direction of the mechanical environment. Thus, the need for technology to translate these mechanical stresses is fundamental to establishing the efficacy for preserving health of tissues in the future.

In summary, the instrumentation presented in this technological report is novel, reliable, accurate, and safe for human tissues. To fully translate technology from the laboratory to human studies will require that experts from engineering, rehabilitation, and biosciences, work collaboratively to advance the field of human regenerative rehabilitation.

## Conclusions

This report presents a novel example of how to deliver compressive and vibratory loads to the lower limb in humans via a new technology. Mechanical loads such as vibration and direct limb load have not been systematically studied in various combinations in humans. Importantly, the vibration stimuli developed in this report is directed to a single limb, rather than to the whole body, allowing a method to compare the direct effects of load to specific tissue. By delivering isolated therapeutic doses of mechanical stress to human tissues, we anticipate that the optimal methods of mechanically and physiologically stressing tissues may be ascertained in future studies.

## Availability and requirements

**Project name:** Human Regenerative Rehabilitation

**Project home page:** Not applicable

**Operating system(s):** Platform independent

**Programming language:** Not applicable

**Other requirements:** Not applicable

**License:** Not applicable

**Any restrictions to use by non**-**academics:** Not applicable

Availability of supporting data

No supporting data will be submitted with this manuscript.

## Abbreviations

SCI: Spinal cord injury; Hz: Hertz; G: Gravitational force of the Earth; kPa: Kilopascal; %BW: Percent body weight; EMG: Electromyography; FFT: Fast Fourier transform; RMS: Root means square; ICC: Intra-class correlation coefficient; VEGF: Vascular endothelial growth factor; H-reflex: Hoffmann reflex; %FS: Percent full scale; DAQ: Data acquisition.

## Competing interests

The authors declare that they have no competing interests.

## Authors’ contributions

CM helped implement the system, collected and analyzed data, and drafted the manuscript. JW provided technical assistance to implement the system and assisted with editing the manuscript. RKS conceived and developed the technology, provided oversight of all testing, procured funding, and edited/ approved the final draft of the manuscript. All authors read and approved the final manuscript.
